# Addiction of pancreatic cancer cells to zinc-finger transcription factor ZIC2

**DOI:** 10.18632/oncotarget.4960

**Published:** 2015-07-22

**Authors:** Shingo Inaguma, Hideaki Ito, Miho Riku, Hiroshi Ikeda, Kenji Kasai

**Affiliations:** ^1^ Department of Pathology, Aichi Medical University School of Medicine, Nagakute, Aichi, Japan

**Keywords:** ZIC2, GLI1, ANXA8, FGFR3, apoptosis

## Abstract

Activity of GLI transcription factors of Hedgehog signaling is key for various cancer cell properties, especially in pancreatic ductal adenocarcinoma (PDAC). Zinc-finger transcriptional regulators *ZIC1* to *ZIC5* of *ZIC* gene family were demonstrated to associate with GLI to increase the nuclear accumulation and transcriptional activity of GLI. Notwithstanding this supportive role for GLI-dependent transcription, it was not fully understood whether ZIC plays an independent role in cancer cell biology. Here, we found that ZIC2 is indispensable in the regulation of PDAC cell apoptosis. We found that human PDAC cell lines uniquely express ZIC2. *ZIC2* knockdown induced PDAC cell apoptosis; conversely, ZIC2 over-expression enhanced the cellular proliferation. Through a comprehensive screening, we identified *fibroblast growth factor receptor 3* (*FGFR3*) and *ANNEXIN A8* (*ANXA8*) as genes up-regulated by ZIC2 in PDAC cells. The forced expression of these two genes cooperatively rescued the apoptosis of *ZIC2*-knockdown cells. Immunohistochemical analyses further supported the correlation of ZIC2 expression and these genes in human pancreata harboring PDAC. Intriguingly, the ZIC2-mediated up-regulation of *FGFR3* and *ANXA8* was indicated to be GLI -independent. This evidence highlights the indispensable role of ZIC2 in regulating cellular proliferation and apoptosis during PDAC development and suggests a potential therapeutic target for PDAC.

## INTRODUCTION

The members of the *ZIC* gene family, vertebrate homologues of the *Drosophila odd-paired* gene, encode a nuclear protein harboring a C2H2 type of zinc-finger domain that shows a notable homology to the GLI transcription factors GLI1-3 of the Hedgehog pathway[1] [2]. ZIC is mainly expressed in the developing or mature central nervous system (CNS) in a spatiotemporally restricted manner, and ZIC2 mutation causes various developmental anomalies, including holoprosencephaly, which is also linked to the mutations of the Hedgehog pathway-related genes [3].

GLI1 and GLI2 are over-expressed and thought to participate in the development and progression of various cancers, especially pancreatic ductal adenocarcinoma (PDAC) [4]. It was experimentally confirmed that GLI1 is indispensable for the oncogenic mutant type of *KRAS*-dependent transformation of pancreatic epithelium in a genetically-modified mouse model [5-7] and that GLI1 is essential for the survival and maintenance of the transformed phenotype of human PDAC cell lines. Indeed, GLI1 was revealed to up-regulate the expression of a variety of genes crucial for many cancer cell properties [8-12].

A previous study using the electrophoretic mobility shift assay (EMSA) and *in vitro* binding analysis reported that GLI and ZIC bound to similar DNA sequences, indicating a close relationship between ZIC and GLI in the regulation of downstream target genes [2]. Moreover, ZIC was reported to associate with GLI and enhance the nuclear accumulation and transcriptional activity of GLI proteins. [13, 14] Therefore, it might be possible that the ZIC-dependent regulation of GLI target genes is involved in the cell properties of PDAC; however, whether ZIC is expressed in PDAC cells and the role of ZIC, if expressed, have not been addressed.

In the present study, we first identified *ZIC2* as a unique member of the *ZIC* gene family expressed in PDAC cells. ZIC2 knockdown lead to PDAC cell apoptosis and, in turn, ZIC2 over-expression enhanced PDAC cell proliferation. We found that ZIC2 up-regulated the expression of *FGFR3* and *ANXA8*, and these two genes cooperatively rescued the apoptotic cell death of *ZIC2*-knockdown cells. Interestingly, we revealed that the up-regulation of *FGFR3* and *ANXA8* by ZIC2 occured in a GLI-independent manner. Our results uncovered the indispensable and GLI-independent role of ZIC2 in the regulation of PDAC cell apoptosis.

## RESULTS

### ZIC2 regulates the apoptotic cell death of PDAC cells

**Figure 1:**
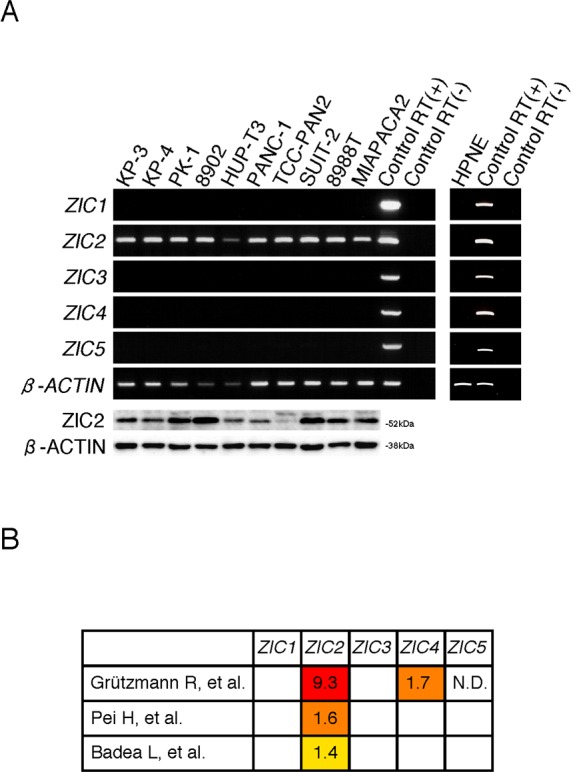
ZIC2 is uniquely expressed in PDAC cells **A**, RT-PCR and immunoblot analysis of the human PDAC cell lines and the immortalized normal pancreatic epithelial cell line HPNE. Normal fetal cerebellar RNAs with or without the RT reaction were used as controls. **B**, The relative expression of the *ZIC* gene family in PDAC tissue normalized to normal tissue. The data sets were drawn from the Oncomine database. The original publications are listed in the references. Note that Grützmann R. et al. [16]. used microdissected samples for cDNA array analysis.

**Figure 2:**
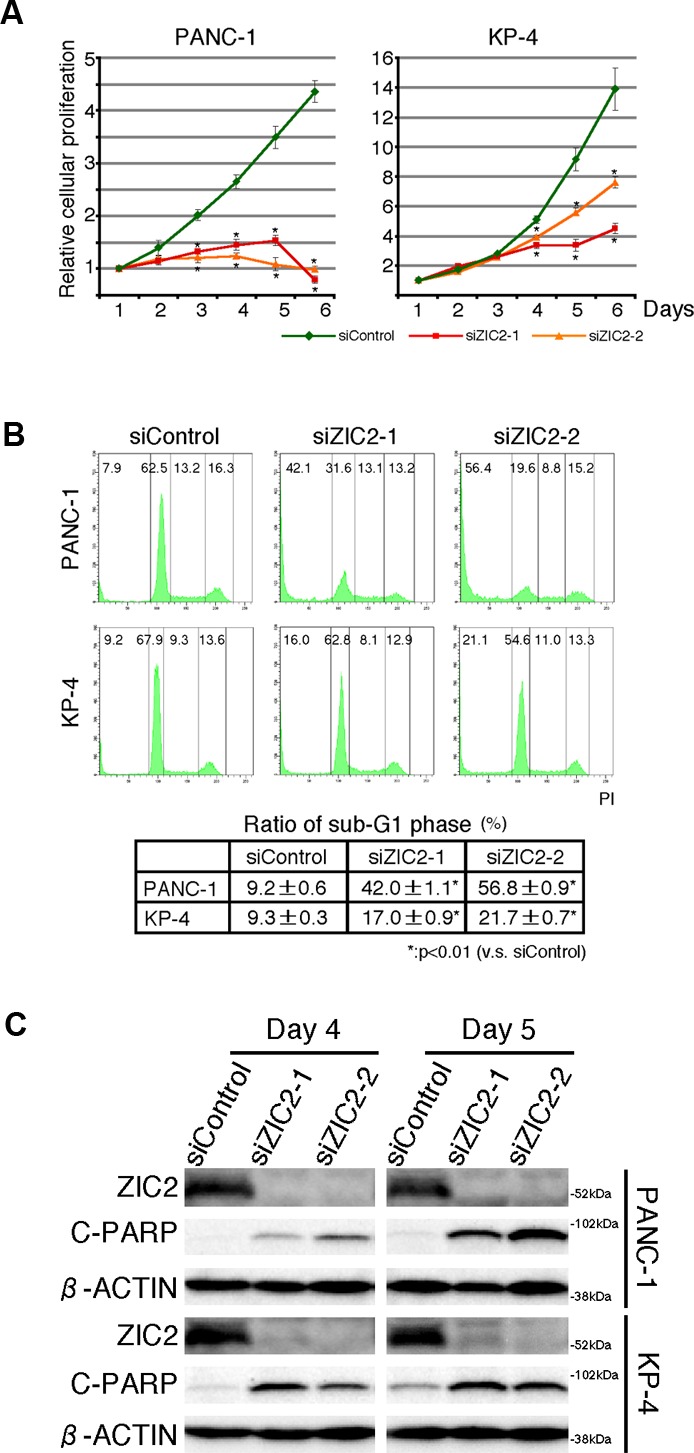
ZIC2 regulates apoptotic cell death of PDAC cells **A** and **B**, The proliferation curves and cell cycle analysis of PDAC cells transfected either *ZIC2*-specific (siZIC2-1, siZIC2-2) or control (siControl) siRNAs. The data represent the mean ± SD from three independent experiments. C, immunoblot analysis of PDAC cells transfected with siRNAs.

We first examined the expression of the *ZIC* gene family (*ZIC1*-*5*) in immortalized normal pancreatic epithelial cells, hTERT-HPNE (HPNE) cells and human PDAC cell lines. Reverse transcription (RT)-PCR analysis revealed that none of the *ZIC* members were expressed in HPNE cells while the expression of all members of the family was detected in a control material (human cerebellum cDNA). We found, however, that all of the human PDAC cell lines we tested uniquely expressed ZIC2 (Figure 1A). Compared with three independent datasets in the Oncomine database [15-17], we again found that *ZIC2* expression was dominantly increased in PDAC cells rather than normal pancreatic tissue (Figure 1B). Therefore, we hypothesized that ZIC2 may be involved in the PDAC development. To clarify this, we examined the effect of *ZIC2* knockdown in the human PDAC cell lines PANC-1 and KP-4 by the transfection of specific siRNA (for knockdown validation, see Figure 2C). We found that the transfection of *ZIC2*-specific siRNA (siZIC2-1 and siZIC2-2) but not control siRNA (siControl) suppressed the cellular proliferation of both cell lines (Figure 2A). Cell cycle analysis revealed that *ZIC2* knockdown reduced the fraction of cells in the G0/G1 phase and increased the fraction of cells in the sub-G1 phase (representative data in Figure 2B, *upper panels*; the statistical analysis from three independent experiments in Figure 2B, *lower table*). Additionally, *ZIC2* knockdown concordantly increased the amount of cleaved PARP (Figure 2C). These results indicated the indispensable role of ZIC2 in regulating the apoptotic cell death of PDAC cells. Next, we analyzed the effects of ZIC2 using newly established cell lines from the human PDAC cell line PANC-1: these derivatives show the Tet-regulated expression (Tet-off) of ZIC2 (Supplementary Figure S1A). As a result, we found that the forced expression of ZIC2 enhanced the G1-S transition and cellular proliferation (Supplementary Figure S1B and C). It was reported that ZIC2 over-expression induces anchorage-independent growth and transformed foci of mouse embryonic fibroblasts (MEF) [18]. Indeed, we also observed that lentiviral-transduced ZIC2 enhanced cellular proliferation (Supplementary Figure S1D) and the anchorage-independent growth of HPNE cells (Supplementary Figure S1E). Unfortunately, neither ZIC2- nor control (LacZ)-transduced HPNE cells were transplantable to NOD/SCID mice in our experimental conditions (data not shown): therefore, these results indicated that ZIC2 over-expression led to the cellular proliferation of the pancreatic duct epithelium, while we could not conclude at this moment that it could transform the pancreatic duct epithelium.

### ZIC2 up-regulates ANXA8 and FGFR3

**Figure 3:**
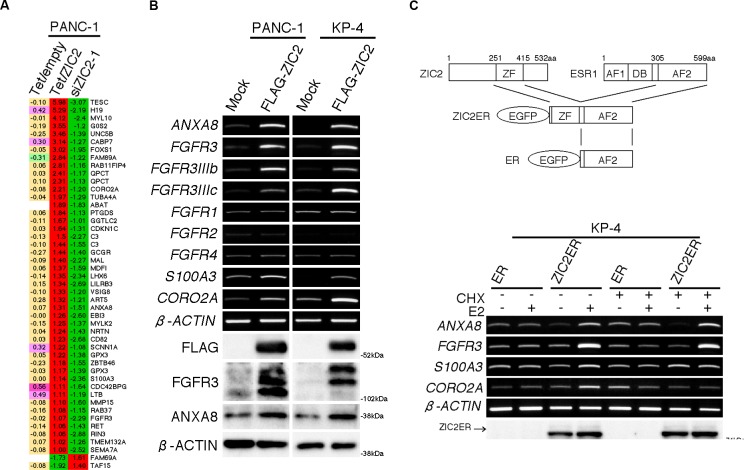
ZIC2 up-regulates ANXA8 and FGFR3 **A**, Comparative presentation of three sets of the expression microarray analysis. Tet/empty, the genes of PANC-1^Tet/empty^ cells not changed by the DOX elimination; Tet/ZIC2, the genes of PANC-1^Tet/ZIC2^ cells changed by the DOX elimination; siZIC2-1, the genes of PANC-1 cells changed by siZIC2-1-treanfection, which was normalized by siControl transfection. The numbers are on the log2 scale. **B**, RT-PCR and immunoblot analyses of PDAC cells transiently transfected with either a FLAG-tagged ZIC2 expression vector or an empty (Mock) vector. **C**, Schematic of the transgenes (*upper panel*) and RT-PCR / immunoblot analysis (*lower panel*) of KP-4 cells, which were transiently transfected with either ZIC2ER or a control ER vector. *CHX*, cycloheximide; *E2*, β-estradiol.

To date, ZIC2 over-expression was reported to impact the clinical course and prognosis of patients harboring ovarian cancer [18], endometrial cancer [19] and oral squamous cell carcinoma [20], while the molecular mechanism of disease progression was not revealed. Given that ZIC2 is a transcription factor, we hypothesized that ZIC2 might regulate the expression of genes that are responsible for PDAC cell proliferation and apoptosis. To investigate this possibility, we performed three sets of expression (cDNA) microarray analyses and identified the differentially-expressed genes as follows: from the first set, the genes changed by *ZIC2* knockdown in PANC-1 cells; from the second set, the genes changed by the elimination of DOX in PANC-1^Tet/ZIC2^ clone1; and from the third set, the genes that were “left unchanged” after the elimination of DOX in control PANC-1^Tet/empty^ to eliminate the artificial change by DOX itself. By comparing the chosen genes from these three independent analyses, we identified 43 up-regulated genes, as well as 2 down-regulated genes, as candidate genes downstream of ZIC2 (Figure 3A; for a validation, Supplementary Figure S2A). From the list, we chose *ANXA8*, *FGFR3*, *S100A3* and *CORO2A* and confirmed that their expression levels were indeed up-regulated by the transient transfection of the FLAG-tagged ZIC2 (FLAG-ZIC2) expression vector in either PANC-1 or KP-4 cells (Figure 3B).

Next, we transiently transfected KP-4 cells with either the chimeric gene *ZIC2ER*, which consisted of the EGFP-tagged DNA-binding (zinc-finger) domain of human *ZIC2* and the AF2 domain of the mouse *Esr1*, or its control EGFP-tagged AF2 domain (*ER*; illustration of the chimeric genes in Figure 3C, *upper panel*). The former, not the latter, was expected to rapidly up-regulate the expression of downstream genes upon treatment with β-estradiol (E2), as we previously reported [9, 10]. Indeed, E2 treatment up-regulated the expression of these four genes only in the *ZIC2ER*-transfected cells (Figure 3C). Furthermore, the up-regulation of *ANXA8* and *FGFR3* but not *S100A3* and *CORO2A* was also observed even in the presence of cycloheximide (CHX), indicating that the up-regulation of *ANXA8* and *FGFR3* was not dependent on new protein synthesis (Figure 3C). We also confirmed the up-regulation of *ANXA8* and *FGFR3* in ZIC2-transduced HPNE cells (Supplementary Figure S2B). This evidence indicated that *ANXA8* and *FGFR3* were downstream of ZIC2.

The *FGFR* gene family of receptor tyrosine kinases is known to regulate the cellular proliferation of a variety of cancers, including PDAC [21]. *ANXA8* is a member of the super gene family encoding calcium- and phospholipid-binding proteins, which participate in various cellular functions, including calcium signaling, vesicle trafficking, cell division, growth regulation and apoptosis [22]. Recently, *ANXA8* was reported to be up-regulated in PDAC tissue [23] and to suppress the apoptotic cell death of PDAC cells [24]. Therefore, we examined *FGFR3* and *ANXA8* as a downstream target of ZIC2 in PDAC cells.

Recently a comprehensive analysis of the ZIC2-binding domain revealed that ZIC2 preferentially binds to a transcriptional enhancer, the majority of which are located outside of the transcription start site [25]. Indeed, we found that ZIC2 did not activate the luciferase reporter constructs harboring up to 4kb DNA fragments of the 5′ flanking sequence from transcription start sites of *FGFR3* and *ANXA8* genes (data not shown), leaving the possibility that the distant enhancer of the *FGFR3* and *ANXA8* genes might be responsible for ZIC2-dependent up-regulation.

The other members of *FGFR*, such as *FGFR1*, *FGFR2* and *FGFR4*, were not changed by FLAG-ZIC2 transfection (Figure 3B). The up-regulation of both *RET* and its ligand *Neurtrin* (*NRTN*) was intriguing (Figure 3A and Supplementary Figure S2A), but the expression of *RET* and its co-receptor *GFRA1* were detected only in a few limited PDAC cell lines (Supplementary Figure S3). Therefore, we considered that the NRTN-RET axis might not be a major pathway in PDAC cells.

### Up-regulation of ANXA8 and FGFR3 is GLI-independent

**Figure 4:**
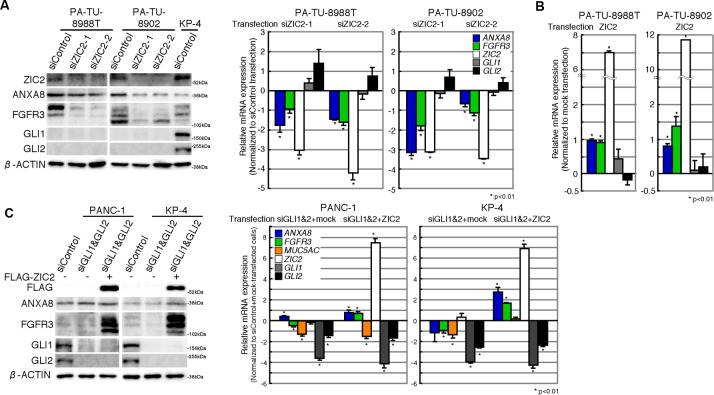
Up-regulation of ANXA8 and FGFR3 is GLI -independent **A**, Immunoblot (*left*) and qRT-PCR (*right*) analyses of GLI low-expressing PDAC cells (PA-TU-8988T and PA-TU-8902) transiently transfected with indicated siRNAs. The immunoblot of GLI high-expressing KP-4 cells is also shown as a comparison. The RT-PCR data were normalized to that of siControl transfectants and are shown on the log2 scale. *Columns*, the mean values of three independent experiments; *bars*, SD. **B**, qRT-PCR analysis of GLI low-expressing PDAC cells transiently transfected with either a FLAG-ZIC2 expression vector or an empty (Mock) vector. The data were normalized to that of mock vector transfectants and are shown on the log2 scale. *Columns*, the mean values of three independent experiments; *bars*, SD. **C**, Immunoblot (*left*) and qRT-PCR (*right*) analyses of GLI high-expressing PDAC cells (PANC-1 and KP-4) transiently transfected with either siRNAs for a GLI1/2-double knockdown (siGLI1&siGLI2) or a control siRNA (siControl) in conjunction with FLAG-ZIC2 (+) or a mock control vector (−). In qRT-PCR analysis, the data were normalized to that of siControl plus a mock control expression vector transfectants and are shown on the log2 scale. *Columns*, the mean values of three independent experiments; *bars*, SD.

It was reported that ZIC2 interacts with GLI1 to enhance the nuclear translocation and transcription activity of GLI1 [13, 14], indicating a supportive role of ZIC2 in GLI1-target gene expression. Thus, we examined whether ZIC2 up-regulates *ANXA8* and *FGFR3* in a GLI-dependent manner. First, we employed human PDAC cell lines PA-TU-8988T and PA-TU-8902, which expressed GLI1 and GLI2 at very low levels (Figure 4A, *left panel*). The expression of *ANXA8* and *FGFR3* was reduced by *ZIC2*-knockdown without an obvious suppression of *GLI1* and *GLI2* (Figure 4A) and was conversely increased by a transient transfection of FLAG-ZIC2 (Figure 4B). Next, using PANC-1 and KP-4, which highly expressed GLI1 and GLI2 (Figure 4C, *left panel*), we found that the *ZIC2* transfection further increased the expression of *ANXA8* and *FGFR3* in the context of double-knockdown of *GLI1* and *GLI2* (Figure 4C). The change of *ANXA8* and *FGFR3* was in contrast to *MUC5AC*, which we previously reported as a direct target gene of GLI1 and GLI2 [9]. This evidence suggested that ZIC2 up-regulates *ANXA8* and *FGFR3* in a GLI-independent manner.

### ZIC2 enhances FGFR3-dependent phosphorylation of ERK

**Figure 5:**
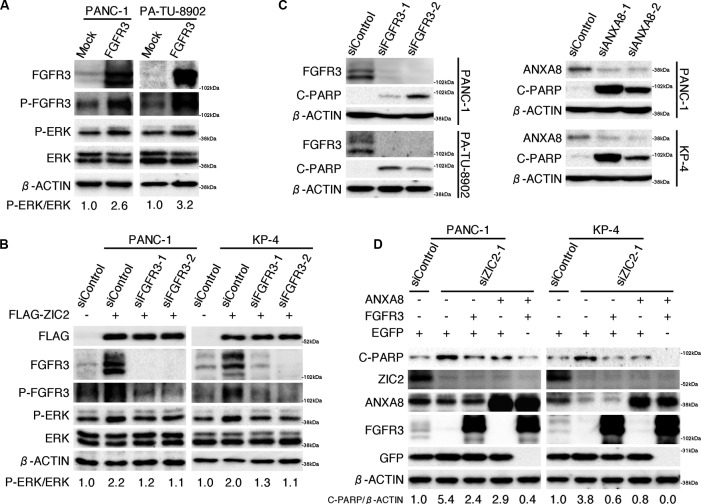
ANXA8 and FGFR3 cooperatively rescue PDAC cells from apoptotic cell death triggered by ZIC2 knockdown **A**, Immunoblot analysis of PDAC cells transiently transfected with either an FGFR3-IIIb variant of the human *FGFR3* expression vector (FGFR3) or an empty (Mock) control vector. The ratio of phosphorylated ERK versus ERK protein was semi-quantified using NIH Image software. **B**, Immunoblot analysis of PDAC cells transiently transfected with either *FGFR3*-specific siRNAs (siFGFR3-1, siFGFR3-2) or a control siRNA (siControl) in conjunction with either FLAG-ZIC2 (+) or an empty (Mock) vector (−). **C**, immunoblot analysis of PDAC cells transiently transfected with either *FGFR3*-specific*, ANXA8*-specific (siANXA8-1, siANXA8-2) or control siRNAs. **D**, Immunoblot analysis of PDAC cells transfected with either siZIC2-1 or siControl in conjunction with the indicated expression vectors (EGFP, FGFR3 and ANXA8). The DNA amounts of transfected expression vectors were equalized using the EGFP expression vector.

While *FGFR1*, *FGFR2* and *FGFR4* are frequently over-expressed in PDAC and are well recognized to enhance cellular proliferation *via* several downstream pathways, including the RAS-MAPK-ERK pathway [21], the effect of FGFR3 in PDAC cells is controversial. Lafitte *et al*. reported that the forced expression of *FGFR3* enhanced the cellular proliferation in a vimentin-rich “mesenchymal” type of PDAC cell *via* the MAPK pathway, while it suppressed the cellular proliferation in an E-cadherin-rich “epithelial” type of PDAC cell [26]. We found that splicing variants of *FGFR3* (“mesenchymal” type PDAC-expressing *FGFR3-IIIb* and “epithelial” type PDAC-expressing *FGFR3-IIIc*) were both up-regulated by FLAG-ZIC2 transfection (Figure 3B). By the transient transfection of the expression vector of *FGFR3-IIIb* splice variant of human *FGFR3* gene, we also found that the FGFR3 up-regulation increased the phosphorylation of FGFR3 itself and ERK in both “epithelial” type PA-TU-8902 and “mesenchymal” type PANC-1 (Figure 5A; Supplementary Figure S4 for *E-cadherin* / *vimentin* expression profile of PDAC cell lines), indicating the impact of FGFR3 on the MAPK pathway in our experimental system.

Next, to examine the effect of ZIC2 on the FGFR3-MAPK pathway, we transiently transfected PANC-1 and KP-4 with the FLAG-ZIC2 expression vector in conjunction with either *FGFR3*-specific siRNAs or a control siRNA. We found that the forced expression of *ZIC2* enhanced the phosphorylation of FGFR3 and ERK, coincident with the increased expression of FGFR3. We also found that the *ZIC2*-enhanced phosphorylation of ERK returned to the original levels by *FGFR3* knockdown (Figure 5B). We cannot say, however, whether the expression of *FGF1* and *FGF2* (Figure 3B), ligands of FGFR, or a “molecular crowding” of FGFR3 might intrinsically stimulate FGFR3 signaling [21], this question should be investigated in the future.

### ANXA8 and FGFR3 cooperatively rescue PDAC cells from apoptotic cell death triggered by ZIC2 knockdown

The activation of the MAPK-ERK pathway has been known to regulate PDAC apoptosis [27]. Indeed, we found that *FGFR3* knockdown increased cleaved PARP levels in both PDAC cell lines (Figure 5C, *left panel*). Recently, *ANXA8* was reported to be up-regulated in PDAC tissue [23] and to suppress PDAC cell apoptosis [24]. In agreement with this result, we also found that *ANXA8* knockdown led to increased cleaved PARP levels (Figure 5C, *right panel*). Given that *ZIC2* knockdown led to apoptotic cell death, we considered the possibility that FGFR3 or ANXA8 prevents the apoptotic cell death induced by *ZIC2* -knockdown. We transiently transfected PANC-1 and KP-4 with the expression vectors for *ANXA8* or *FGFR3* in conjunction with siRNA for *ZIC2* -knockdown. We found that the forced expression of either *ANXA8* or *FGFR3* alone could slightly but not completely reduce the cleaved PARP induced by *ZIC2*-knockdown, while co-transfection of these two genes showed a better suppression of cleaved PARP (Figure 5D). This evidence suggested that ZIC2 over-expression regulated apoptotic cell death of PDAC cells through the up-regulation of ANXA8 and FGFR3.

### ZIC2 expression correlates with ANXA8 and FGFR3 expression and Ki-67 labeling index in pancreatic tissue

**Figure 6:**
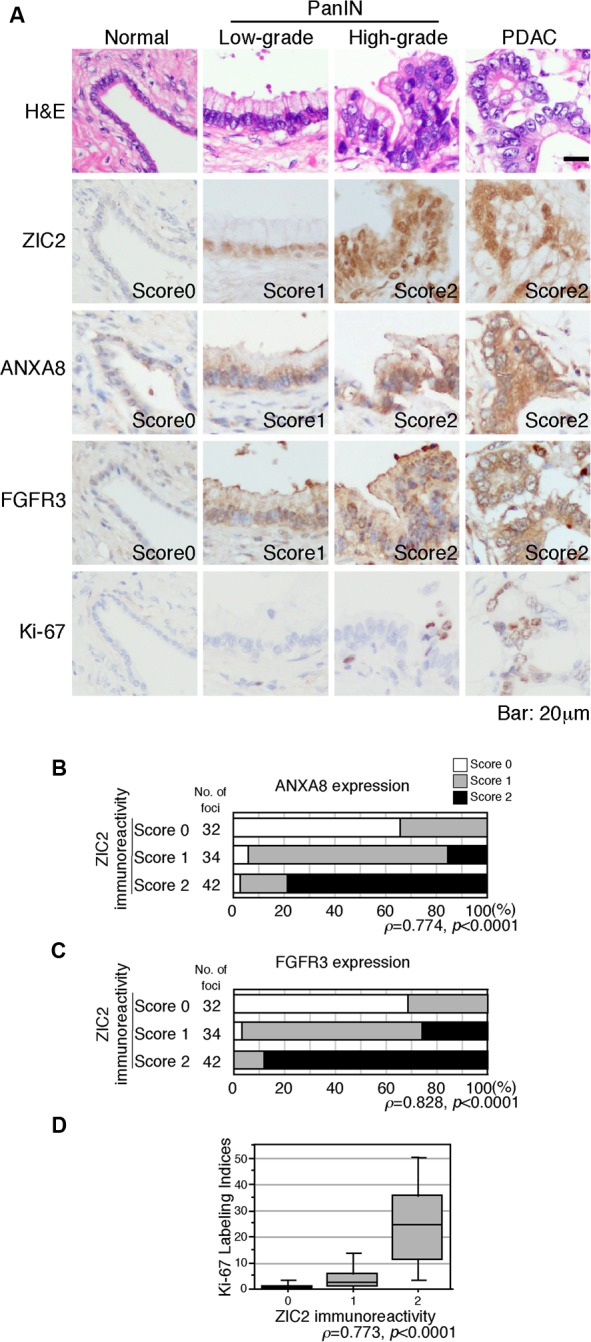
Immunohistochemical analysis of the pancreas harboring PDAC **A**, H&E and immunohistochemical staining of precancerous pancreatic intraepithelial lesion (PanIN) and PDAC. Staining intensities of ZIC2, ANXA8 and FGFR3 were semi-quantitatively scored as negative, 0; weak, 1; strong, 2. Labeling index of Ki-67 staining was calculated by counting over 100 cells per lesion. *Bar*, 20μm. Statistical analyses are provided in Supplementary Tables S1-S4. **B** and **C**, Correlation between ZIC2 and ANXA8 (B) or FGFR3 (C) expression. D, Correlation between ZIC2 expression and the Ki-67 labeling index.

Finally, we examined the expression of ZIC2, ANXA8, FGFR3 and the cell proliferation marker Ki-67 in PanIN and PDAC by immunohistochemistry. To evaluate the expression of ZIC2, ANXA8 and FGFR3, we used a semi-quantitative scoring of the staining intensity on a three-tiered scale (negative, 0; weak, 1; strong, 2) and statistical analysis as previously shown [9, 10]. ZIC2 was not detectable in the normal pancreatic duct, but was faintly expressed in the low-grade PanIN, then increased in the high-grade PanIN and PDAC cells (Figure 6A and Supplementary Table S1). The expression levels of ANXA8 and FGFR3 were also faintly detectable in the normal duct; however, their expression gradually increased along with ZIC2 expression (Figure 6A and Supplementary Tables S2 and S3). Spearman's rank correlation analysis revealed positive correlations between ZIC2 and its target genes (Figure 6B, 6C). The Ki-67 labeling index was 0.5-3% in the normal duct and low-grade PanIN; then it increased to 9-19% and 34% in the high-grade PanIN and PDAC, respectively (Figure 6A and Supplementary Table S4). Spearman's rank correlation analysis also revealed positive correlations between ZIC2 expression and Ki-67 labeling index (Figure 6D).

## DISCUSSION

The dysregulated expression of GLI1 and GLI2 plays a crucial role in the development and progression of many types of human cancers, including PDAC [5-7]. To fully activate the transcription activity of GLI, a multistep molecular mechanism is required [28]: for instance, we reported that the *SIL* / *STIL*-mediated derepression from Suppressor-of-Fused is involved during PDAC development [29]. It was previously reported that ZIC and GLI bind to similar DNA sequences *in vitro* [2] and that ZIC enhances the nuclear accumulation and transcriptional activity of GLI proteins [13, 14], suggesting a supportive role for ZIC in the activation of the GLI target expression. However, knowledge regarding the role of ZIC in cancer is limited: ZIC1 was reported as a tumor -suppressor in gastric [30] and colorectal cancers [31] because of its down-regulated or silenced expression due to the promoter hypermethylation, while another group showed that ZIC1 is over-expressed and plays an oncogenic role in liposarcoma [32]. ZIC2 expression was revealed to correlate with a worse clinical course of ovarian cancer [18] and oral cancer patients [20], while the molecular mechanism of ZIC2 expression in adult tumor cells and worse prognosis were not fully understood.

In the present study, we revealed that ZIC2 is uniquely expressed in PDAC cells and regulated the cellular proliferation and apoptotic cell death of PDAC cells through the up-regulation of *FGFR3* and *ANXA8* expression. Furthermore, using GLI high- and low-expressing PDAC cell lines, we revealed that the up-regulation of those genes occured in a GLI-independent manner. This evidence highlights the unique role of ZIC2 in the regulation of PDAC cell apoptosis.

In a variety of human malignancies, FGFR signaling is activated by several molecular mechanisms [21]. For instance, some cases of bladder cancer harbor the mutated *FGFR3* gene, leading to the ligand-independent dimerization or enhanced kinase activity of FGFR3 [28]. In the present study, we found that the forced expression of *ZIC2* induced the up-regulation of both splice variants of *FGFR3* (*FGFR3-IIIb* and *IIIc*) (Figure 3B) and that *FGFR3* knockdown induced apoptotic cell death in both the “epithelial” and “mesenchymal” types of PDAC (Figure 5C). Therefore, we currently speculate that a physiological or “*ZIC2*-inducible” level of FGFR3 might contribute the regulation of apoptotic cell death, even in the “epithelial” type of PDAC, or that the combined up-regulation with *ANXA8* can prevent the suppressive effect of overexpressed FGFR3 on the cell proliferation of the “epithelial” type of PDAC.

*ANXA8* belongs to a vertebrate “A subgroup” of the annexin superfamily coding a calcium- and membrane-binding protein. The “A subgroup” consists of at least twelve members (A1-A11and A13), all of which are suspected to be involved in tumor development [22]. *ANXA8* has been reported to be up-regulated in BRCA1-related breast cancer [33] and PDAC [23]. It was also revealed that that the forced expression of *ANXA8* increased the BrdU incorporation of PANC-1 cells and *ANXA8* knockdown conversely induced apoptotic cell death [22]; however, the regulatory mechanism of *ANXA8* expression is unknown. Here, we showed that *ANXA8* as well as *FGFR3* were downstream of ZIC2 in PDAC cells. Unfortunately, we failed to identify the ZIC2-binding site(s) within up to 4kb DNA fragment of the 5′ flanking sequence from transcription start sites of the *ANXA8* and *FGFR3* genes. Recently, Luo *et al*. performed ZIC2 chromatin immunoprecipitation and sequence (ChIP-seq) analysis in mouse ES cells and revealed that ZIC2 occupied enhancers, the majority of which, however, did not overlap with a transcription start site and instead resided within a gene or upstream / downstream of the gene [25]. This report might suggest the possibility of the presence of the ZIC2-binding site(s) outside of the genomic region that we examined in the present study.

To the best of our knowledge, the mechanism(s) underlying ZIC2-overexpression in cancer cells is currently unknown, which should be investigated in the future.

In conclusion, our evidence uncovered the indispensable and unique role of ZIC2 in PDAC in regulating cell proliferation and apoptotic cell death *via* GLI-independent downstream genes, underlining ZIC2 as a potential therapeutic target in PDAC patients.

## MATERIALS AND METHODS

### Cells, plasmid vectors and siRNAs

The immortalized normal pancreatic epithelial cells, hTERT-HPNE cells, were purchased from the ATCC. The origins of the other human PDAC cell lines and the methods for their authentication were previously described [10]. PANC-1^Tet/ZIC2^ clone1, clone2 and their control PANC-1^Tet/empty^ were established from human PDAC cell line PANC-1 using a tetracycline-regulated (Tet-off) system according to the manufacturer's instructions (Clontech). Doxycycline (DOX) was used as a regulator of the Tet-off system. The expression vectors for the chimeric gene *ZIC2ER* and its control *ER*, *ANXA8* and *FGFR3* were generated by PCR -amplification and cloning into the pcDNA 3.1 expression vector (Invitrogen). The plasmid vector for the construction of FLGA-ZIC2 and LacZ-expressing lentivirus (CSII-CMV-MCS-IRES2-Bsd) was kindly gifted by Dr. Hiroyuki Miyoshi (RIKEN BRC, Japan). To knock -down *ZIC2*, *ANXA8* and *FGFR3*, 21-nucleotide duplex siRNAs were synthesized as follows (Nippon Gene, Japan): siZIC2-1, 5′-GAAGAGCUGCAACAAAACUTT-3′ and 5′-AGUUUUGUUGCAGCUCUUCTT-3′; siZIC2-2, 5′-GUGCGAGUUUGAGGGCUGUTT-3′ and 5′-ACAGCCCUCAAACUCGCACTT-3′; siANXA8-1, 5′-CCCAAAACCUCCACAGCUATT-3′ and 5′-UAGCUGUGGAGGUUUUGGGTT-3′; siANXA8-2, 5′-AGGAGGGUGUCAUCAUUGATT-3′ and 5′-UCAAUGAUGACACCCUCCUTT-3′; siFGFR3-1, 5′-CACCCUACGUUACCGUGCUTT-3′ and 5′-AGCACGGUAACGUAGGGUGTT-3′; and siFGFR3-2, 5′-ACUGCACACACGACCUGUATT-3′ and 5′-UACAGGUCGUGUGUGCAGUT-3′. The following duplexes were used as a control: siControl 5′-GACAACGACGAAAGAUACUTT-3′ and 5′-AGUAUCUUUCGUCGUUGUCTT-3′. The siRNAs for GLI1 (siGLI1) and GLI2 (siGLI2) were previously reported [34].

### Antibodies and real-time PCR analyses

The antibodies used were as follows: anti-ZIC2 antibody (ARP35821, Aviva Systems Biology); anti-GLI2 antibody (sc-271786; Santa Cruz Biotechnology), anti-p-FGFR-3 (Tyr724) antibody (sc-33041, Santa Cruz Biotechnology); anti-GLI1 L42B10 antibody, anti-FGFR3 C51F2 antibody, anti-p44/42 MAPK 137F5 antibody, anti-phospho-p44/42 MAPK 20G11 antibody, anti-cleaved PARP D64E10 antibody (Cell Signaling); anti-FLAG M2 antibody, anti-β-actin AC-74 antibody (SIGMA); and anti-ANXA8 antibody (GTX103853, GenTex). The methods for the real-time PCR (qRT-PCR) analyses were previously described [10]. All data were analyzed in triplicate.

### cDNA microarray analysis

Total RNA was purified using the RNeasy mini kit (Qiagen) from PANC-1^Tet/ZIC2^ clone1 and PANC-1^Tet/empty^ before and 48 hours after DOX elimination. Total RNA was also extracted from PANC-1 cells transfected with either siZIC2-1 or siControl siRNA for 96 hours. Gene expression was analyzed using Agilent 4x44K cDNA microarray (Agilent Technologies) according to the manufacturer's protocol. Microarray data are available from the NCBI Gene Expression Omnibus (GEO) database (GSE39704).

### Cell proliferation and FACS analysis

To quantify cell numbers, Cell Titer 96(R) Aqueous One Solution (Promega) was used. Cell cycle distributions were analyzed using a Click-iT® EdU Alexa Fluor® 488 Flow Cytometry Assay Kit (Life Technologies) or propidium iodide (PI) staining. The stained cells were counted using FACSCanto II flow cytometer (BectonDickinson). All data were analyzed in triplicate.

### Anchorage-independent growth analysis

Lentiviral-transduced HPNE cells were cultured in a soft -agar. The number of cells in the soft -agar was measured using a Cytoselect 96-well cell transformation assay (Cell Biolabs Inc., USA) according to the manufacturer's protocol.

### Immunohistochemical analysis

Twenty PDAC cases were selected for the study based on the availability of tissue samples from the archives of the Department of Pathology at the Aichi Medical University Hospital. Sample use was approved by the Institutional Ethical Review Board. Serial sections from formalin-fixed, paraffin-embedded tissue samples were subjected to the hematoxylin and eosin (H&E) staining and immunohistochemical staining. The staining intensities of ZIC2, ANXA8 and FGFR3 were semi-quantitatively scored using a three-tiered scale and statistically analyzed using a Mann–Whitney U test. For the analysis of Ki-67 staining, a labeling index was calculated by counting at least one hundred of cells in the lesion. The correlations between ZIC2, ANXA8, FGFR3 expressions and Ki-67 labeling index were estimated using Spearman's correlation coefficient. P-values of <0.05 were considered statistically significant. StatView 5.0 (SAS Institute Inc.) was used for statistical analyses.

## SUPPLEMENTARY MATERIAL FIGURES AND TABLES


