# Supplementary material for: Addiction of pancreatic cancer cells to zinc-finger transcription factor ZIC2
Source: Oncotarget. 2015 Jul 22;6(29):28257–68. doi: 10.18632/oncotarget.4960 (PMC4695058; doi:10.18632/oncotarget.4960)
Supplement: Supplementary file 1 [file oncotarget-06-28257-s001.pdf]

Addiction of pancreatic cancer cells to zinc-finger transcription factor ZIC2

Supplementary Material

Supplementary Figure S1

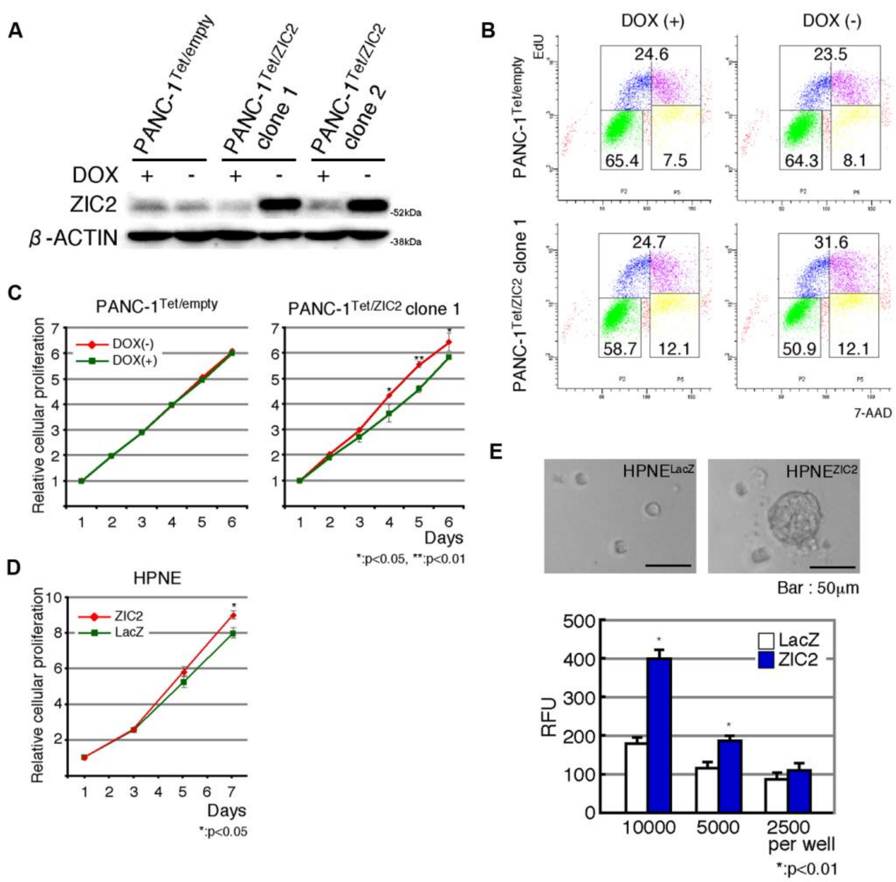

Supplementary Figure S1

(A) Immunoblot analysis of Tet-inducible PANC-1 cell lines.

(B) Cell cycle analysis of Tet-inducible PANC-1 cell lines.

(C) Cell proliferation analysis of Tet-inducible PANC-1 cell lines.

(D) Cell proliferation analysis of lentiviral-transduced HPNE cells.

(E) Anchorage-independent cel growth of HPNE cells. *Upper*, representative photos of lentiviral-transduced HPNE cells cultured on the soft agar for 21 days. *Lower*, cell numbers were measured using CytoSelectTM 96-well Cell transformation Assay kit (CELL BIOLABS, INC., CA).

Supplementary Figure S2

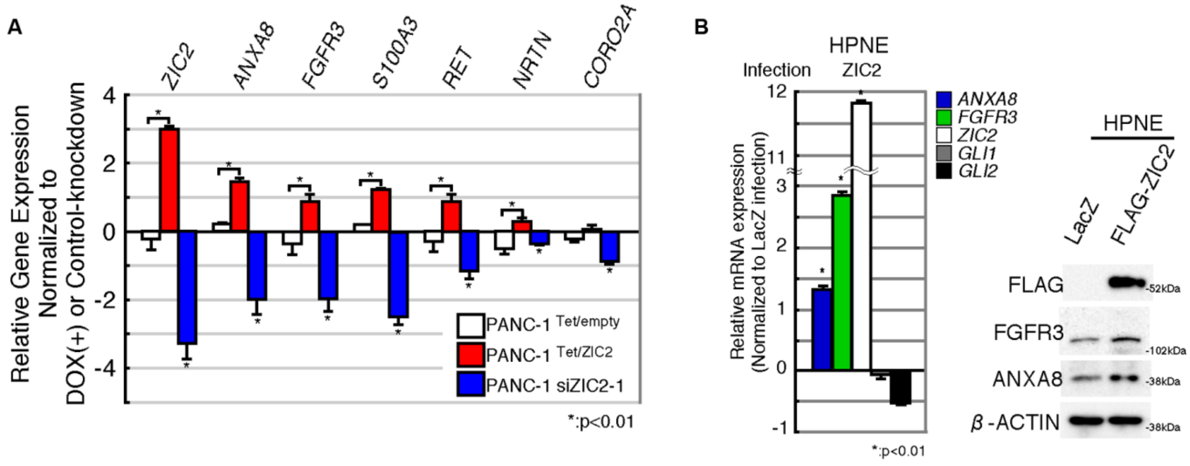

Supplementary Figure S2

(A) qRT-PCR analysis for a validation of the microarray analysis.

(B) qRT-PCR analysis (*left*) and immunoblot analysis (*right*) of HPNE cells, in which either FLAG-ZIC2 or a control LacZ were lentivirally transduced.

Supplementary Figure S3

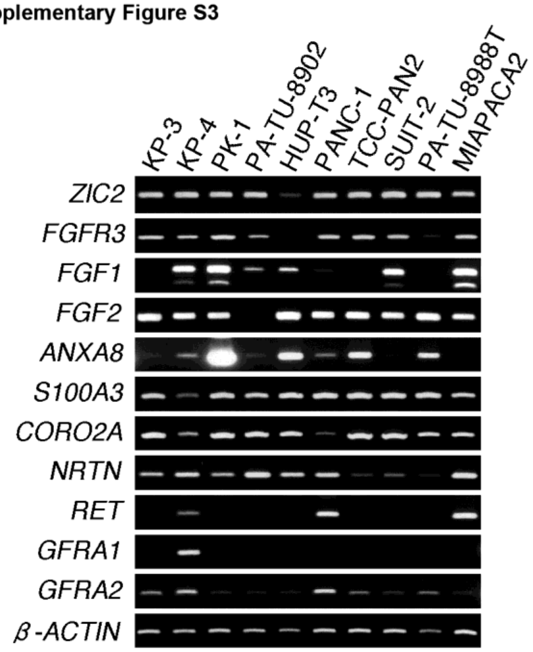

Supplementary Figure S3

RT-PCR analysis of human PDAC cell lines

Supplementary Figure S4

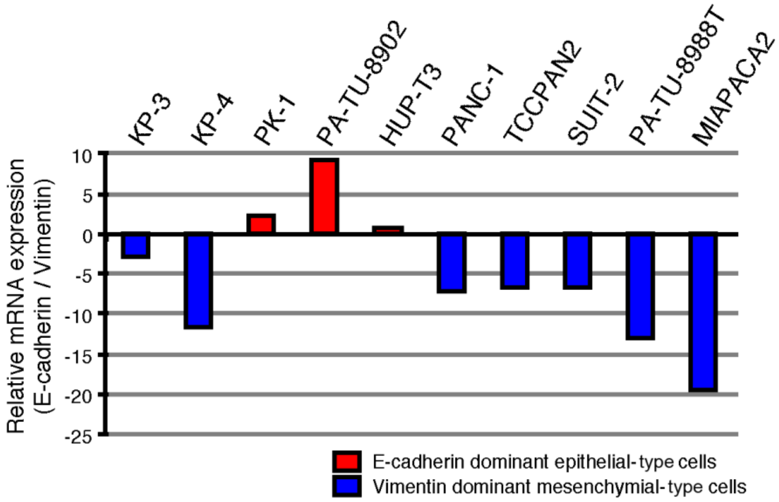

Supplementary Figure S4

qRT-PCR analysis of human PDAC cell lines.

Supplementary Table S1. ZIC2 expression in Pancreatic Lesions

	Number of foci	ZIC2 immunoreactivity			*P* value
		Score 0	Score 1	Score 2	
Normal	26	23 (88.5)	3 (11.5)	0 (0)	-
PanIN-1A	15	6 (40.0)	7 (46.7)	2 (13.3)	<0.01
PanIN-1B	17	2 (11.8)	14 (82.4)	1 (5.9)	<0.01
PanIN-2	14	1 (7.1)	5 (35.7)	8 (57.1)	<0.01
PanIN-3	10	0 (0)	2 (20.0)	8 (80.0)	<0.01
PDAC	26	0 (0)	3 (11.5)	23 (88.5)	<0.01

Mann-Whitney *U* test vs Normal

Supplementary Table S2. ANXA8 expression in Pancreatic Lesions

	Number of foci	ANXA8 immunoreactivity			*P* value
		Score 0	Score 1	Score 2	
Normal	26	16 (61.5)	10 (38.5)	0 (0)	-
PanIN-1A	15	5 (33.3)	9 (60.0)	1 (6.7)	=0.10
PanIN-1B	17	1 (5.9)	14 (82.4)	2 (11.8)	<0.01
PanIN-2	14	1 (7.1)	8 (57.1)	5 (35.7)	<0.01
PanIN-3	10	1 (10.0)	3 (30.0)	6 (60.0)	<0.01
PDAC	26	0 (0)	1 (3.8)	25 (96.2)	<0.01

Mann-Whitney *U* test vs Normal

Supplementary Table S3. FGFR3 expression in Pancreatic Lesions

	Number of foci	FGFR3 immunoreactivity			*P* value
		Score 0	Score 1	Score 2	
Normal	26	18 (69.2)	8 (30.8)	0 (0)	-
PanIN-1A	15	3 (20.0)	8 (53.3)	4 (26.7)	<0.01
PanIN-1B	17	1 (5.9)	12 (70.6)	4 (23.5)	<0.01
PanIN-2	14	1 (7.1)	7 (50)	6 (42.9)	<0.01
PanIN-3	10	0 (0)	3 (30.0)	7 (70.0)	<0.01
PDAC	26	0 (0)	1 (3.8)	25 (96.2)	<0.01

Mann-Whitney *U* test vs Normal

Supplementary Table S4.
Ki-67 Labeling Index in Pancreatic Lesions

	Number of foci	Ki-67 Labeling Indices	*P* value
Normal	26	0.46 ± 0.75	-
PanIN-1A	15	0.98 ± 0.69	<0.05
PanIN-1B	17	3.25 ± 1.41	<0.01
PanIN-2	14	8.70 ± 4.15	<0.01
PanIN-3	10	18.79 ± 6.32	<0.01
PDAC	26	33.92 ± 9.54	<0.01

t-test vs Normal
